# A new species of *Scelidopetalon* Delkeskamp (Coleoptera, Erotylidae) from China with a key to world species of the genus

**DOI:** 10.3897/zookeys.317.5112

**Published:** 2013-07-19

**Authors:** Cong-Chao Dai, Mei-Jun Zhao

**Affiliations:** 1 Department of Biology, College of Life and Environmental Sciences, Shanghai Normal University, Shanghai, 200234, P. R. China

**Keywords:** Coleoptera, Erotylidae, *Scelidopetalon*, *Pseudamblyopus* identification key, new record genus, new species, China

## Abstract

A new species *Scelidopetalon biwenxuani* **sp. n.** is described from China, representing the first occurrence of the genus in Hainan province. A key to the world species of this genus is provided.

## Introduction

The subfamily Erotylinae includes colorful fungus-feeding beetles commonly called “pleasing fungus beetles”. They are worldwide in distribution with over 2500 described species. In general, species of the tribe Tritomini are characterized by an antennal club of 3 to 5 antennomeres, dilated maxillary palpi, closed procoxal cavities (with prosternal-proepimeral suture located at the midpoint posterior to the procoxae), meso-metasternal suture having a broadened dicondylic connection, and pseudotetramerous tarsi (tarsomere IV small and hidden by an expanded tarsomere III). Larvae and adults feed on larger basidiomycete fungi (e.g. mushrooms, polypore or bracket fungi, etc.). The name “pleasing fungus beetle” is likely derived from the large size and colorful patterns of many species.

**Figures 1–2.**
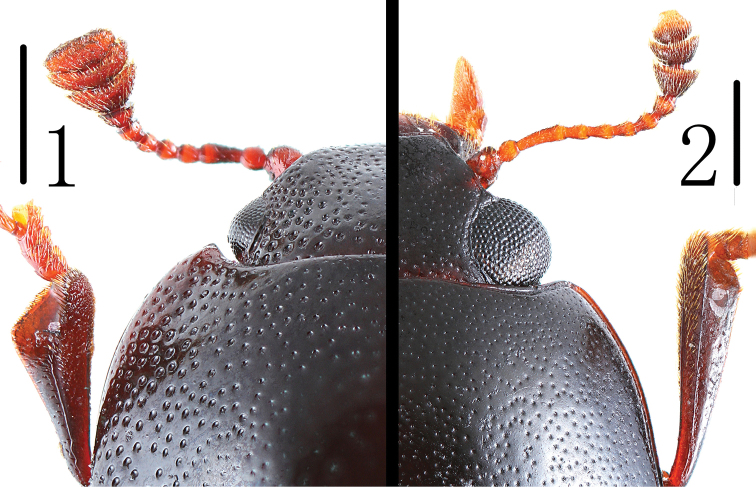
Head of **1** *Scelidopetalon biwenxuani* and **2** *Amblyopus vittatus* in dorsal view. Scale = 0.5 mm.

While examining Burmese specimens of *Amblyopus* Lacordaire, Gorham (1896) established *Petaloscelis* based on its small and finely facetted eyes (Fig. 1), which are large and coarsely facetted in *Amblyopus* (Fig. 2). Arrow (1925, 1926) described four additional species in *Petaloscelis*. While examining the African species, Arrow (1945) noted that Gorham confused African species with an Indian species and considered *Petaloscelis* a synonym of *Amblyscelis* Gorham. Delkeskamp (1957) thought *Petaloscelis* and *Amblyscelis* were distinct based on eye characters as mentioned above and on differences in tibial dilation. Because the name *Petaloscelis* Gorham was a junior homonym of *Petaloscelis* Bergoth (see Chûjô and Chûjô 1990), Delkeskamp (1957) proposed the name *Scelidopetalon* for the species of *Petaloscelis* Gorham with *Petaloscelis instabilis* Gorham, 1896, as the type.

Araki (1941) described *Pseudamblyopus* for the Japanese species *Amblyopus palmipes* Lewis. Araki noted *Pseudamblyopus* is easily distinguished from *Amblyopus* by the small and finely facetted eyes and mentioned this genus differs from *Petaloscelis* in the antennal club structure. But neither Arrow nor Delkeskamp were aware of Araki’s work. Thus, *Scelidopetalon* and *Pseudamblyopus* were both separated from *Amblyopus* based on the same characters.

Because no specimens of *Pseudamblyopus* are available for study,we are not treating *Scelidopetalon* as a synonym to *Pseudamblyopus*. This should be considered in future revisionary work.

Previously, a total of six species have been reported for *Scelidopetalon* and two species for *Pseudamblyopus* worldwide.

*Scelidopetalon instabilis* (Gorham 1896) (Burma, Viet-Nam)

*Scelidopetalon similis* (Arrow 1925) (Assam Valley)

*Scelidopetalon varicolor* (Arrow 1925) (India)

*Scelidopetalon solidus* (Arrow 1925) (India)

*Scelidopetalon fasciatus* (Arrow 1926) (Sumatra, N. Borneo)

*Scelidopetalon arrowi* Delkeskamp (1957) (Singapore)

*Pseudamblyopus palmipes* (Lewis 1889) (Japan)

*Pseudamblyopus similis* (Lewis 1887) (Far East, Japan)

In this work, one new species of *Scelidopetalon* is described and illustrated: *Scelidopetalon biwenxuani* sp. n. from Hainan Province, China.

## Material and methods

To examine the genitalia, the abdominal segments were detached from the body after softening in hot water. The genitalia, together with other dissected parts, were mounted in Euparal (Chroma Gesellschaft Schmidt, Koengen, Germany) on plastic slides. Photos of sexual characters were taken with a FUJIFILM X10 camera attached to an Olympus SZX 16 stereoscope; habitus photos were taken with a Canon macro photo lens MP-E 65 mm attached to a Canon EOS7D camera.

The specimen treated in this study is deposited in the following public collection:

SNUCDepartment of Biology, Shanghai Normal University, P. R. China

## Taxonomy

### *Scelidopetalon* Delkeskamp and *Pseudamblyopus* Araki

**Diagnosis.** These two generacan be distinguished from other Tritomini genera by eyes small and finely facetted (Fig. 1). Tibiae (Fig. 8) triangular, with the extremities very broad and hollowed for the tarsi. Prosternal lines of prosternum (Fig. 7) short, not extending in front of procoxal cavities. These three characters have not existed together in other genera of Tritomini. The distinct difference between species of these two genera is antennal club structure. Most species of *Scelidopetalon* with antennomere XI much broader than long, one species of *Scelidopetalon* and all the species of *Psudodamblyopus* with antennomere XI almost as long as broad.

### Key to world species of genus *Scelidopetalon* and *Pseudamblyopus*

Parts of the following key were taken from Arrow (1925).

**Table:** 

1	Antennomere XI almost as long as broad	2
–	Antennomere XI much broader than long	4
2	Pronotum yellow, elytra with rather indefinite reddish patch at base	*Scelidopetalon varicolor* (Arrow)
–	Pronotum orange, elytra without rather indefinite reddish patch at base	3
3	Legs black	*Pseudamblyopus palmipes* (Lewis)
–	Legs brown	*Pseudamblyopus similis* (Lewis)
4	Elytra with markings	5
–	Elytra without markings	8
5	Pronotum black	*Scelidopetalon biwenxuani* Dai & Zhao, sp. n.
–	Pronotum orange to red	6
6	Pronotum with a black median line	*Scelidopetalon fasciatus* (Arrow)
–	Pronotum without a black median line	7
7	Pronotum with black spots, elytra with X-shaped orange markings	*Scelidopetalon instabilis* (Gorham)
–	Pronotum without black spots, elytra with orange markings not X-shaped	*Scelidopetalon arrowi* Delkeskamp
8	Dark above	*Scelidopetalon similis* (Arrow)
–	Pale above and beneath	*Scelidopetalon solidus* (Arrow)

#### Scelidopetalon biwenxuani

Dai & Zhao sp. n.

urn:lsid:zoobank.org:act:B6AA8AE6-86AE-4B7D-A172-CC01ED471933

http://species-id.net/wiki/Scelidopetalon_biwenxuani

Figs 1 3–4 5–9

##### Type material.

**Holotype:** **CHINA: Hainan Prov.:** 1♀, Jianfengling N.R., Mingfenggu Valley, 18°44'N, 108°50'E, alt. 1000 m, 15.V.2011, Bi Wen-Xuan leg. (SNUC).

##### Description.

**Figures 3–4.**
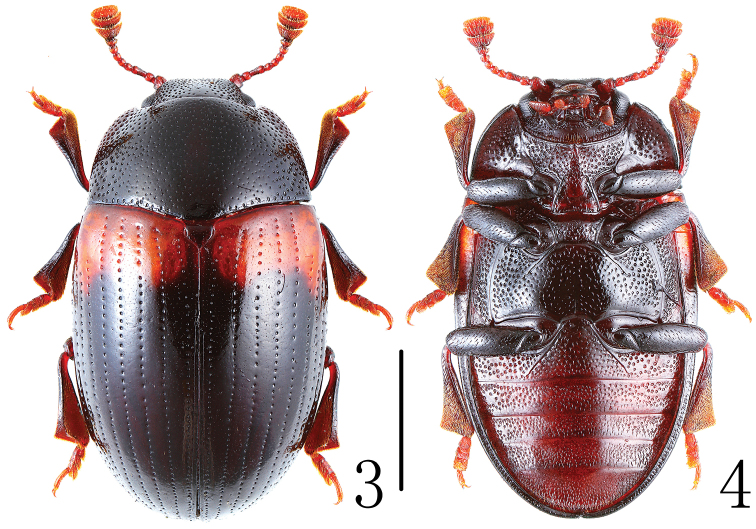
Habitus of *Scelidopetalon biwenxuani* in dorsal and ventral view. Scale = 2 mm.

Body (Figs 3, 4) oval, convex, shining; length: 6.50 mm; width: 3.79 mm. Body black; legs, palpi and antennae reddish-brown. Elytron black, with red at basal third.

**Figures 5–9.**
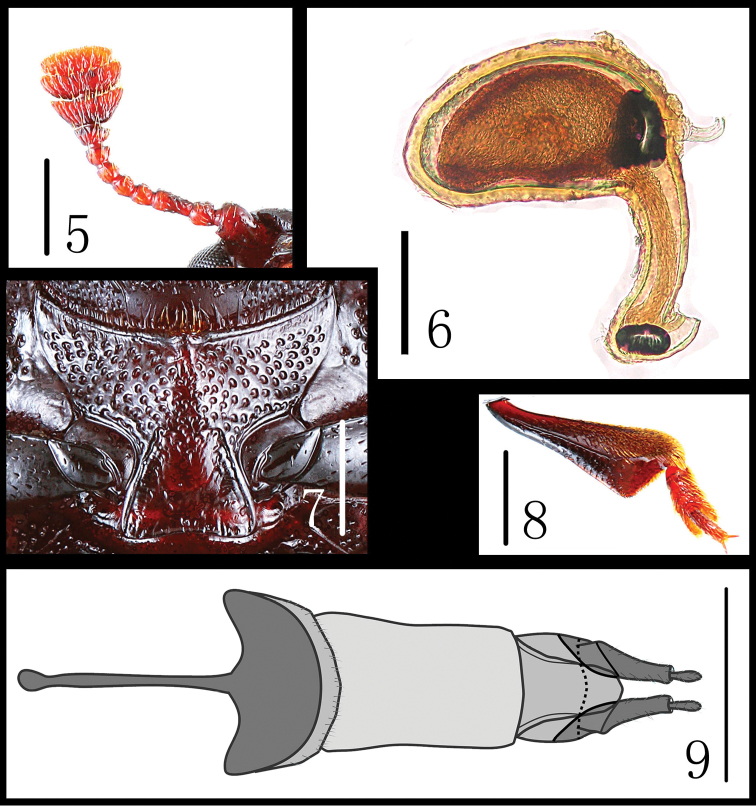
*Scelidopetalon biwenxuani*. **5** antenna **6** spermatheca **7** prosternum **8** tibia and tarsus **9 **female genitalia. Scales = 0.5 mm (**5, 7, 9**), Scales = 0.1 mm (**6**), Scales = 1.0 mm (**9**).

Head (Fig. 1) width between eyes = 5.5 times eye diameter in dorsal view; punctation coarse, separated by 0.5–1.0 puncture diameters laterally and 2–4 puncture diameters medially; stridulatory files not evident. Antennae (Fig. 5) short, not extending behind posterior border of pronotum; antennomere III about 2.0 times as long as IV; antennomeres IV to VIII short; antennomeres IX to XI broad and transverse; relative lengths of antennomeres II–XI: 9.0: 14.0: 7.0: 7.0: 7.0: 7.0: 6.5: 9.0: 9.0: 8.0. Maxillary terminal palpomeres trapezoidal, 1.67 times wider than long. Mentum broad with anterior projection, almost pentagonal, 1.75 times wider than long.

Pronotum transverse, convex above, widest at base (pl/pw = 0.70); anterior angles weakly projecting; lateral margins gently curved toward eyes. Pronotum distinctly punctured medially, finely and closely punctured laterally.

Prosternum (Fig. 7) strongly punctured, the front margin is produced to a short point in the middle, prosternal lines short, not extending in front of procoxal cavities; Mesosternum coarsely punctured. Metasternum coarsely punctured at the sides and almost smooth in the middle, with distinct coxal lines. Abdomen fairly strongly and closely, its sides coarsely, punctured, with distinct coxal lines on first ventrite nearly attaining posterior margin.

Scutellum pentagonal, finely and sparely punctured.

Legs short, tibiae triangular, with the extremities very broad and hollowed for the tarsi.

Elytra widest at middle, then gradually narrowing to apex; each with 9 punctate lines.

Female genitalia (Fig. 9) with gonostyli fringed apically with some elongate setae; female spermatheca (Fig. 6) with capsule almost egg-shaped.

##### Distribution.

China (Hainan Province).

##### Diagnosis.

The new species can be distinguished from other species in this genus by the black pronotum and red markings of the elytra.

##### Etymology.

This species is named in honor of Mr. Wen-Xuan Bi, collector of the new species.

## Supplementary Material

XML Treatment for Scelidopetalon biwenxuani
